# Premium Offered

**Published:** 1865-10

**Authors:** 


PREMIUM OFFERED.—Dr. T. C. Brinsmade of Troy, N. Y., offers a premium of $100 for the best essay on medical and vital statistics, with a plan for hosptal reports and records of private practice, and a draft of a registration law. The essay is to be handed to the Committee of the New York State Medical Society on Prize Essays by the 15th of December next.

